# Explainable Mammogram Analysis with EfficientNetV2 and Grad-CAM++ for Robust Cancer Diagnosis

**DOI:** 10.3390/diagnostics16010105

**Published:** 2025-12-28

**Authors:** Mohammed Ameen

**Affiliations:** Department of Information Systems, Faculty of Computing and Information Technology, King Abdulaziz University, Rabigh 21911, Saudi Arabia; mkameen@kau.edu.sa

**Keywords:** breast cancer, EfficientNet V2, CBAM, CLAHE, explainable artificial intelligence, Grad CAM++

## Abstract

**Background:** Breast cancer remains a leading cause of mortality among women worldwide, underscoring the need for timely and accurate detection. Conventional mammographic diagnosis, while widely used, is limited by subjectivity and variability in interpretation. Recent advances in deep learning (DL) have improved automated detection; however, the black-box nature of these models raises concerns regarding clinical trust and interpretability. **Methods:** To address this, we propose an explainable DL framework for breast cancer classification using mammographic images. The approach employs contrast limited adaptive histogram equalization (CLAHE)-based preprocessing to enhance lesion contrast, EfficientNetV2 for feature extraction, and the convolutional block attention module (CBAM) to refine salient features. For interpretability, gradient-weighted class activation mapping++ (Grad-CAM++) is used to highlight discriminative regions influencing predictions. **Results:** The framework is evaluated on three publicly available datasets—MIAS, DDSM, and InBreast—individually and under cross-dataset settings. Results demonstrate superior performance over existing methods, achieving classification accuracies of 99.85%, 99.40%, and 99.70% on MIAS, DDSM, and InBreast, respectively, with corresponding F1-scores of 99.75%, 99.10%, and 99.55%. Confusion matrix analysis confirms excellent sensitivity for malignant cases, and time complexity assessments show reduced training and inference overhead compared to conventional deep models. **Conclusions:** The framework thus provides a robust and interpretable solution for mammogram-based breast cancer screening.

## 1. Introduction

Breast cancer continues to be the most commonly diagnosed cancer in women and a significant contributor to cancer-related mortality worldwide [1,2]. It was responsible for approximately 2.3 million new cases and over 665,000 fatalities in 2022 [3]. The estimates suggest that there will be a 38% increase in cases and a 68% increase in deaths by 2050. This increase will be primarily felt by low-human development index (HDI) countries [4]. Improving survival outcomes, facilitating timely detection, and reducing dependence on invasive procedures necessitate early and precise detection.

The standard screening instrument for the identification of breast abnormalities and tumors is mammography which is a low-dose X-ray imaging technique [5]. However, there are certain factors that impact its efficacy, such as inter-observer variability, dense breast tissue, and atypical lesion presentations. This poses a persistent challenge in achieving consistent and reliable interpretation.

Conventional computer-aided diagnosis (CAD) systems have been developed to aid radiologists in analyzing mammograms. These systems generally rely on handcrafted features such as texture, shape, and edge descriptors. These features are subsequently exploited by shallow classifiers such as support vector machines (SVM), decision trees (DT), or random forests (RF) [6]. Nevertheless, these methodologies have numerous limitations such as their sensitivity to noise, manual feature engineering requirement, lack of robustness to variations in imaging conditions and patient demographics. Moreover, handcrafted features may inadequately represent complex patterns in high-resolution medical pictures, hence limiting the diagnostic efficacy of conventional CAD systems [7].

The emergence of deep learning (DL), especially convolutional neural networks (CNNs), has revolutionized breast cancer detection through mammographic images. CNNs may autonomously learn hierarchical features from unprocessed picture data, thereby negating the necessity for manual feature engineering [8,9]. Various CNN-based architectures, including ResNet, DenseNet, and EfficientNet, have been investigated for breast cancer classification tasks [10,11]. These models surpass conventional techniques by effectively capturing both low-level and high-level visual indicators related to malignancy. Yet DL models often operate as black box entities, complicating the comprehension of the reasoning underlying their predictions [12]. The absence of transparency constitutes a significant obstacle to their clinical implementation.

In the realm of medical diagnostics, particularly in cancer diagnosis, elucidation is essential. Physicians must justify a diagnosis and interpret model outputs according to clinical evidence. The recent rise in research on explainable artificial intelligence (XAI) seeks to address this issue by enhancing the interpretability and reliability of machine learning models [13]. Methods include gradient-weighted class activation mapping (Grad-CAM++) [14], layer-wise relevance propagation (LRP) [15], and local interpretable model-agnostic explanations (LIME) [12] have arisen as effective instruments for visualizing the components of an image that impact a model’s decision. In mammography, these approaches may reveal suspicious areas in breast tissue, allowing radiologists to validate and comprehend model predictions.

Although several XAI approaches are available in the literature, an agreement on how to incorporate them into computationally efficient diagnostic systems remains a challenge. Numerous existing techniques are either computationally intensive, relying heavily on large labeled datasets, or have weak generalization capability across diverse imaging sources. Moreover, some recent studies focus solely on performance metrics while inadequately validating interpretability, which is essential in critical medical domains.

To deal with these issues, a comprehensive, interpretable system for multi-class classification of mammographic images is proposed in this study that integrated advanced CNN architectures and attention-augmented interpretability methods. To enhance the visibility of subtle lesion features prior to model training, contrast limited adaptive histogram equalization (CLAHE) is applied for preprocessing [16]. Furthermore, EfficientNetV2 [17] is employed for feature extraction, the convolutional block attention module (CBAM) for attention enhancement [18], and Grad-CAM++ [19] for visual elucidation of predictions. The model is individually trained and validated on three well-known public mammography datasets: mammographic image analysis society dataset (MIAS) [20], digital database for screening mammography (DDSM) [21,22], and InBreast [23,24]. All datasets undergo uniform preprocessing and data augmentation to guarantee uniformity, improve contrast, and replicate acquisition variability.

Recent research has sought to include XAI into breast cancer classification frameworks. For instance, ref.  [19] introduced a hybrid approach in which model transparency is improved by integrating shapley additive explanations (SHAP) with CNN model. Although effective, the model lacked attention-guided refinement and used old CNN architecture that may not enhance system efficiency. Another study in [25] used LIME to elucidate logistic regression outputs for tabular symptom datasets. which is less relevant in intricate image-based diagnostics. On the other hand, the proposed research in this paper integrates advanced and light-weight DL model with efficient attention mechanisms and visual elucidations to guarantee both precision and clarity across various datasets. The major contributions of this research are as follows:

- A novel breast cancer classification pipeline is created by integrating CLAHE preprocessing, EfficientNetV2 for feature extraction, CBAM for attention refinement, and Grad-CAM++ for visual interpretability. This pipeline enables high accuracy and transparency in decision-making.
- The framework is consistently implemented across three publicly accessible mammographic datasets (MIAS, DDSM, InBreast), thereby guaranteeing consistency through standardized preprocessing and augmentation and validating model robustness through individual and cross-dataset evaluations.
- The integration of channel and spatial attention through CBAM enhances feature localization and emphasizes clinically pertinent regions, resulting in superior classification performance with minimal false negatives, particularly for malignant cases.
- Grad-CAM++ generates class-specific heatmaps that provide visual explanations that are radiologically meaningful, thereby bridging the distance between the outputs of AI models and clinical comprehension. They also maintain a low computational overhead, ensuring real-time usability.

The remainder of this paper is organized as follows: Section 2 reviews the existing literature on traditional and explainable approaches for breast cancer detection. Section 3 presents the proposed methodology, including data preprocessing, model architecture, and explainability strategy. Section 4 details the experimental setup and provides a comprehensive performance analysis. Finally, Section 5 concludes the paper with key findings and future directions.

## 2. Literature Review

In recent years, breast cancer detection has undergone a transformation from conventional machine learning methods to more sophisticated DL and explainable AI approaches. Although conventional methods heavily depended on handcrafted features and shallow classifiers, they frequently lacked robustness and were unable to generalize across a variety of imaging datasets. By explicitly learning hierarchical representations from raw mammograms, the emergence of CNNs has significantly enhanced classification accuracy. Nevertheless, the interpretability and clinical trust of these DL models are frequently questioned due to their tendency to operate as black boxes. XAI techniques have been incorporated into diagnostic frameworks to improve transparency and decision validation in order to overcome these constraints. This section examines the development of breast cancer detection methodologies, emphasizing the advantages and disadvantages of both conventional machine learning and XAI-based DL models.

### 2.1. Traditional Machine Learning-Based Detection Approaches

Classifiers such as SVMs and Random Forests were employed in the early detection of breast cancer, with handcrafted features such as texture and shape. Although moderately effective, these models were constrained by their reliance on manual feature engineering, sensitivity to noise, and poor generalization.

Previous methodologies for breast cancer screening mainly depended on conventional ML techniques that relied on manual feature extraction. Methods including SVMs, DTs, and RFs were utilized on retrieved texture, shape, and statistical features from mammograms [26]. Although these models shown moderate performance in constrained environments, they were dependent on manually crafted features, inadequate scalability across diverse datasets, and sensitivity to noise. These constraints prevented its use in complex diagnostic environments where nuanced and fluctuating patterns are essential.

A DenseNet-based approach incorporated with attention mechanisms was proposed [27]. The method improved classification performance across a variety of mammographic datasets by utilizing multi-level transfer learning. Although enhanced feature localization and generalization were achieved, it involves increased computational complexity. The FCCS-Net framework presented in [28], employed a fully convolutional attention mechanism across multiple levels of a pre-trained ResNet18 to improve the focus of features in breast cancer histopathology images. The method exhibited robust performance on the BreakHis, IDC, and BACH datasets and enhanced the representation of inter- and intra-channel features. However, its generalizability to other imaging modalities was restricted due to its dependence on histopathological data.

In [29], three transfer learning models, MobileNetV2, ResNet50, and VGG16, were merged with LSTM and features were extracted from ultrasound images (USIs). Class imbalance issues were handled through the synthetic minority over-sampling technique (SMOTE) with Tomek (SMOTETomek). An ultrasound-based breast cancer classification system, ODTLD-CABCC, was introduced in [30]. The framework integrated two-level preprocessing, ResNet101-based feature extraction, and a Sailfish Optimizer-tuned LWELM model for classification. The method exhibited enhanced accuracy in classification, segmentation, and noise reduction. However, its scalability to larger datasets or real-time applications may be restricted by the complexity of the optimization process and its reliance on extensive parameter tuning.

Custom and hybrid DL models specifically tailored for mammographic classification have also been presented in recent research. The Hybrid CNN model presented in [31] merged handcrafted and learned features and reported robust performance on the MIAS and DDSM datasets. IMPA-ResNet50 [32] extended the base ResNet50 model with input and middle-path attention mechanisms to enhance feature discrimination. ResNet50 + FPO [33] added frequency positional encoding to enhance texture feature extraction in breast tissue. BreastNet-SVM [34] integrated convolutional feature extraction with a support vector machine classifier, providing robustness in class imbalance handling in DDSM. An All-Conv Net model [35] also removed fully connected layers to prevent overfitting and used only convolutional operations and was tested on InBreast.

### 2.2. Advances in Explainable Deep Learning for Mammography

With the growing need on model transparency, XAI methods have been incorporated into breast cancer detection frameworks. Grad-CAM, SHAP, and LIME techniques have been pivotal in providing visual explanations and enhancing clinical trust. In [36], an attention-guided Grad-CAM method was proposed using infrared breast images. An ensemble of three pre-trained networks was created and Grad-CAM visualizations were incorporated into it to emphasize concerning areas in mammograms, enhancing clinical interpretability; however, the model was deficient in advanced attention refinement for more precise interpretations.

A SHAP-based feature attribution method using gene expression data was proposed in [37] to improve interpretability in breast cancer diagnosis. The study provided robust feature-level explanations but concentrated primarily on structured gene expression data and also did not address spatial information inherent in imaging-based diagnostics. Another work [38] introduced a lightweight CNN integrated with LIME to provide localized interpretability for early breast cancer prediction. Despite its computing efficiency, LIME’s reliance on local perturbations may result in unstable interpretations in high-dimensional medical imaging.

A hybrid architecture utilizing XAI was suggested in [39]. It integrated both image and numerical characteristics for breast cancer classification. A U-Net-based transfer learning model was employed for picture segmentation and prediction, while an ensemble model combined a CNN model with Random Forest and SVM. Although the architecture efficiently utilized multimodal inputs, the study did not provide a thorough assessment of the influence of interpretability on clinical decision-making. In [40], the authors introduced a two-stage architecture, XAI-MethylMarker, for breast cancer classification using DNA methylation data. An autoencoder was used for dimensionality reduction and a feed-forward neural network for subtype prediction. In the second stage, various XAI techniques were applied to identify an interpretable biomarker set. While the framework effectively reduced biomarker complexity, it is limited to non-imaging genomic data and lacks spatial diagnostic insight. An ML-based decision support system for cancer prediction with the incorporation of explainability via SHAP was presented in [41]. Various classifiers were evaluated with XGBoost achieving the highest performance. SHAP analysis was used to interpret feature contributions to the prediction. However, the approach was limited to tabular data. Table 1 summarises the previous methods included in the literature review.

In addition to CNN-based architectures, several recent studies have explored transformer-based encoders for breast cancer diagnosis using full-field digital X-ray mammograms. For example, the ETECADx framework employs an ensemble self-attention transformer encoder on full-field digital mammography, leveraging multi-head self-attention to model long-range dependencies in breast tissue and improve classification performance [42]. Similarly, a hybrid workflow based on residual convolutional blocks followed by a transformer encoder has been proposed for breast cancer classification using digital X-ray mammograms, combining local feature extraction with global contextual modeling [43]. While these transformer-based pipelines demonstrate strong diagnostic accuracy on digital X-ray data, they primarily emphasize architectural advances and classification performance, with comparatively limited focus on explicit, radiologist-oriented visual explanations or cross-dataset robustness analysis. In contrast, the present work adopts an efficient CNN backbone with CBAM-driven attention refinement and Grad-CAM++ visualizations, explicitly targeting interpretable lesion localization and systematic evaluation across multiple heterogeneous mammography datasets.

Prior research has either focused on improving classification accuracy without developing adequate interpretability strategies or using an existing post hoc explanation method without incorporating attention-based feature analysis or cross-dataset testing for robustness. Optimization-intensive architectures incur an additional processing cost. The earliest models of convolutional neural networks do not provide spatial explanations necessary for validating use in clinical settings. We propose a framework that fills these gaps by combining an efficient backbone (EfficientNetV2), two levels of attention refining through CBAM for better localizing lesions, and generating fine-grained visual explanations from Grad-CAM++ while validating performance on three different datasets that differ significantly.

Despite the significant progress made in both traditional and DL-based breast cancer classification systems, there are still significant challenges that need to be addressed. Conventional machine learning models are characterized by inadequate feature representation and limited scalability, whereas numerous DL approaches prioritize accuracy over transparency. However, existing explainable AI techniques, despite their potential, frequently lack integration with high-performing lightweight CNNs and fail to validate interpretability across diverse, high-resolution mammographic datasets. Additionally, the combined effect of attention refinement and class-specific visual explanation in directing model focus toward clinically pertinent regions is typically disregarded in prior research. By proposing a unified, computationally efficient, and explainable DL framework that guarantees both diagnostic performance and clinical trustworthiness, this study endeavors to address these gaps.

## 3. Proposed Methodology

**Figure 1:**
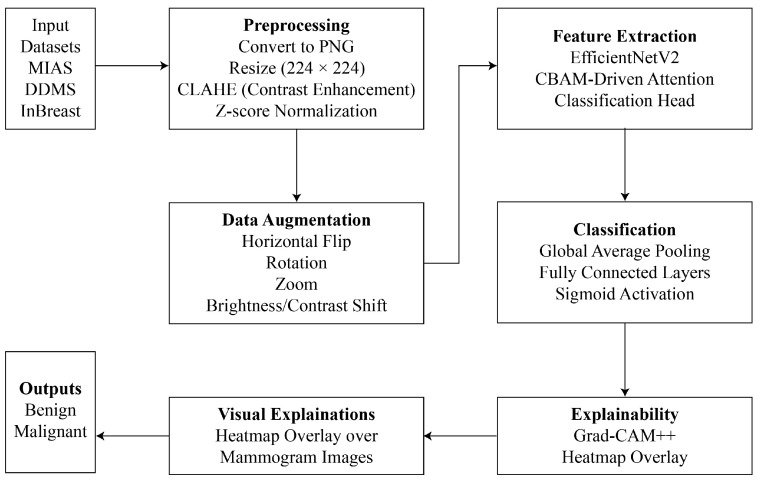
Workflow Diagram of the Proposed Explainable DL Framework.

This study introduces an explainable DL framework that integrates attention mechanisms, visual explanation tools, and advanced CNN architectures to overcome the interpretability and efficiency constraints of automated breast cancer diagnosis. The proposed method comprises systematic preprocessing, real-time augmentation, robust feature extraction with EfficientNetV2, and interpretable predictions with Grad-CAM++. Each component is intended to improve diagnostic accuracy, while simultaneously guaranteeing clinical relevance and transparency. The proposed methodology has been shown in Figure 1. Each phase involved during solution formulation is demonstrated below.

### 3.1. Benchmark Mammography Datasets

This research uses three publicly released mammographic image databases: MIAS [44], DDSM [34], and InBreast [45], differing in image quality, resolution, and clinical heterogeneity.

- MIAS: MIAS consists of 322 grayscale mammogram images of 161 patients, each with left and right breast views. It is annotated with abnormalities like masses and calcifications as well as class labels (malignant or benign). It is low in resolution (normally 1024 × 1024) but heavily used for benchmarking purposes.
- DDSM: DDSM is a large dataset containing more than 2500 cases with digitized mammograms and expert-validated ground truths. It contains image-level labels, lesion segmentation masks, and BI-RADS scores, making it applicable to both classification and detection problems.
- InBreast: InBreast is a full-field digital mammography dataset with high-resolution images, including 410 images from 115 cases. It provides pixel-level lesion annotations, such as masses, calcifications, and architectural distortions, making it suitable for assessing model performance on contemporary, high-quality scans.

**Figure 2:**
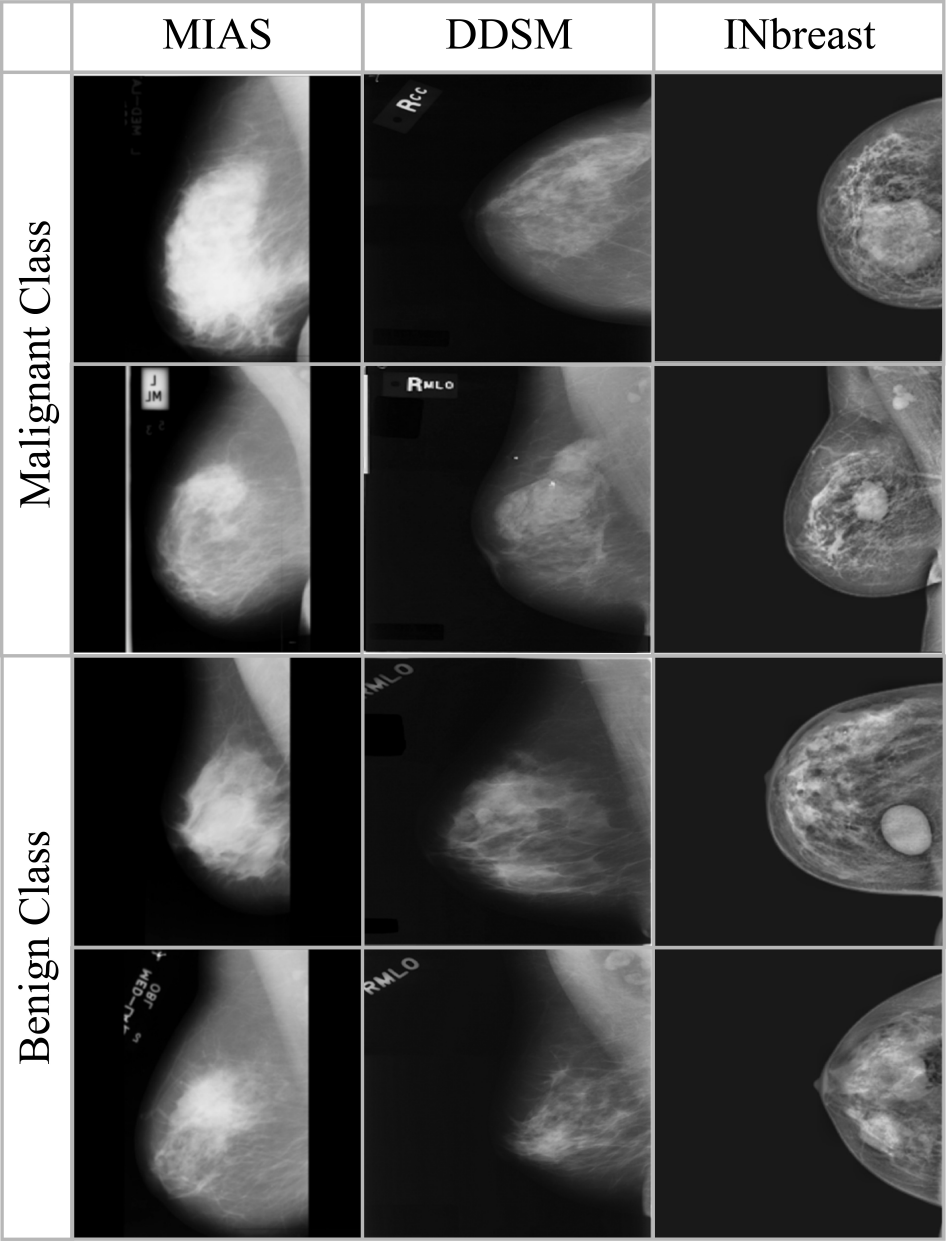
Sample Mammogram Images from MIAS, DDSM, and InBreast Datasets.

Sample images from the three datasets are shown in Figure 2, where as the summary of images in each dataset is presented in Table 2. All the datasets are preprocessed to ensure consistency in image size and intensity distribution. There is no patient overlap between the datasets to allow unbiased assessment across various imaging sources.

### 3.2. Image Preprocessing and Normalization

The uniformity among the diverse datasets is achieved by transforming all mammograms into PNG format and scaling to a fixed resolution of 224×224 pixels with bilinear interpolation [46]. The contrast of the mammograms is enhanced by CLAHE, which increases local contrast and highlights potential lesion regions without adding noise. CLAHE segments the image into small contextual areas (tiles) and applies histogram equalization in each tile. Then a pre-defined clip limit is employed to contrast amplification. This prevents over-enhancement of noise. The contrast-corrected tiles are eventually integrated by bilinear interpolation. This is highly effective in medical imaging, since localized intensity variations sometimes obscure significant results. In mammography, CLAHE refines lesion boundaries and improves subsequent feature extraction without introducing noise. In order to stabilize training and ensure consistent scaling across different datasets, all intensity values of images are normalized using Z-score normalization using Equation (1).

(1) $$z=\frac{x-\mu }{\sigma }$$

where *x* denotes the original pixel intensity, μ and σ are mean and standard deviation of the pixel intensities in the training dataset respectively. Augmentation is an essential step in DL for medical imaging, especially when datasets are limited in size and diversity [47]. In the proposed work, real-time data augmentation is applied during the training. This has enabled to improve model generalization and simulate real-world acquisition variability. Let *I* denote an input mammogram. A transformation *T*, randomly sampled from a distribution of clinically safe operations, is employed such that:

(2) $$\tilde{I}=T\left(I\right),\;T∼U\left(T_{1},T_{2},\ldots ,T_{n}\right)$$

Each $T_{i}$ in the transformation set $\left\{T_{1},T_{2},\ldots ,T_{n}\right\}$ refers to a medically valid variation, as described below:

- Horizontal Flip ($\theta_{\mathrm{flip}}\in \left\{0,1\right\}$)—models left/right breast symmetry.
- Rotation ($\theta_{\mathrm{rot}}\in \left[-10^{\circ },10^{\circ }\right]$)—represents minor deviations in imaging angle.
- Zoom ($\theta_{\mathrm{zoom}}\in \left[0.9,1.1\right]$)—accounts for varying image scale and magnification.
- Brightness/Contrast Shift ($\theta_{\mathrm{bright}}\in \left[0.85,1.15\right]$)—models intensity fluctuations resulting from acquisition parameters.

**Figure 3:**
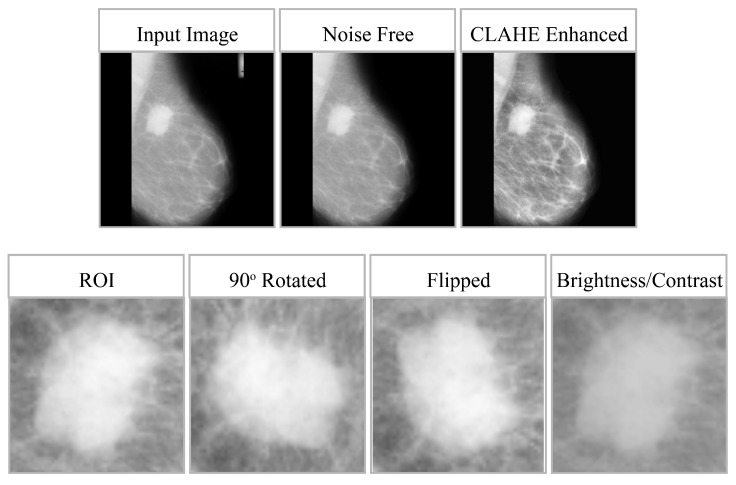
Effect of CLAHE and Data Augmentation on Mammogram Quality.

These augmentations are introduced dynamically during training to enhance robustness, improve the model’s ability to generalize, and reduce overfitting without compromising the anatomical integrity of lesions. The impact of preprocessing and data augmentation is shown in Figure 3, whereas Table 3 provides the effect of data augmentation on all three datasets. For clarity, model training and evaluation were conducted using the full mammographic images only. The regions of interest (RoIs) shown in Figure 3 were manually cropped from the lesion annotation area solely for visualization of the CLAHE enhancement effects and were not used as training inputs. It is important to clarify that the model was trained strictly on full-resolution mammogram images without applying any region-of-interest (RoI) cropping, lesion masking, or patch extraction during training or inference. The small RoI crops shown in Figure 3 were generated solely for qualitative visualization of the CLAHE enhancement and augmentation effects. These RoIs were manually extracted from the dataset’s annotation regions to illustrate localized contrast improvement, but they were never used as inputs to the model. All experiments, including preprocessing, feature extraction, and classification, were performed exclusively on the complete mammographic images.

### 3.3. EfficientNetV2-Based Feature Representation

In this work, EfficientNetV2-Small is used to extract high-level representations from mammographic images. EfficientNetV2 is suitable for resource-constrained medical imaging tasks due to its accuracy and computational efficiency. It employs a compound scaling technique to balance the depth, width, and resolution of the network. The network is initially trained on ImageNet and is fine-tuned on mammographic images to adapt its filters for domain-specific textures and lesion patterns.

The input image $I\in R^{224\times 224\times 1}$ is passed through the network and an intermediate feature representation $F\in R^{H\times W\times C}$ is produced. The resulting feature map preserves both global spatial hierarchies and localized visual patterns. These are then passed to the attention mechanism and the classifier for final prediction. The feature extraction process is defined as

(3) F=E(I)

where E represents the EfficientNetV2 extractor, and $F\in R^{H\times W\times C}$ denotes the resulting deep feature map. These features are subsequently refined through attention mechanisms before being fed into the classification layers.

### 3.4. CBAM-Driven Attention Refinement

A CBAM is applied to further refine the extracted features before classification. CBAM optimizes the model so that only relevant image regions are focused. This is accomplished by a two-step process: initially employing Channel Attention to emphasize the most informative feature channels, followed by spatial attention to emphasize relevant regions in the image. Consequently, an attention-weighted feature representation is obtained which is mathematically represented as

(4) $$F_{\mathrm{att}}=\mathrm{SA}\left(\mathrm{CA}\left(F\right)\right)\cdot F$$

where CA and SA show the channel and spatial attention modules, respectively, and “·” denotes element-wise multiplication. Throughout the overall architecture of the framework proposed above, instead of adding a CBAM module at different locations throughout the EfficientNetV2 architecture’s terminal layers of feature maps, we simply add one single module directly to the terminal convolutional feature maps produced by EfficientNetV2. To achieve this goal, channel and spatial attention mechanisms are applied in that order. Applying channel attention reweights those feature channels that are most discriminative based on descriptors derived from global average pooling and max pooling layer outputs of the EfficientNetV2 network. Spatial attention highlights both salient lesion regions in terms of their respective spatial locations as well as saliency across these locations. Once the feature representation has been enhanced, it is passed onto the global average pooling layer and generates the final output classification via a dense classifier.

### 3.5. Classification Head Architecture

The feature map $F_{att}$ undergoes Global Average Pooling (GAP) to reduce the spatial dimensions. As a result, a vector $v\in R^{C}$ is generated, where each element represents a channel-level summary to convert it into a concise feature vector *v*. This vector captures the most prominent channel-level characteristics throughout mammography. The vector is processed through two dense layers, with the final output layer employing a sigmoid activation function to obtain a multi-class classification score.

### 3.6. Optimization and Learning Parameters

The proposed DL framework for multi-class classification of mammographic images employs the categorical cross-entropy (CCE) loss function, consistent with the experimental training configuration:

(5) $$L_{\mathrm{CCE}}=-\sum_{c=1}^{C}y_{c}\log\left({\hat{y}}_{c}\right)$$

where the variable $y_{c}\in \left\{0,1,2\right\}$ reflects the actual class label (with 0 indicating normal; and 1 benign and 2 malignant) while $\hat{y_{c}}\in \left[0,1,2\right]$ indicates the predicted probability generated by the model.

Next, Adam optimizer is applied to reduce the loss. The optimizer incorporates the advantages of the adaptive gradient algorithm (AdaGrad) with root mean square propagation (RMSProp) and dynamically modifies the learning rate for each parameter. The estimates of the first and second moments of the gradients are used. This approach facilitates stable and efficient convergence. In this study, the optimizer uses the default values, which are: α=0.001, $\beta_{1}=0.9$, $\beta_{2}=0.999$, and $\epsilon =10^{-8}$ as suggested in the original implementation. The weights are adjusted using the following formulation:

(6) $$\theta_{t+1}=\theta_{t}-\alpha \cdot \frac{{\hat{m}}_{t}}{\sqrt{{\hat{v}}_{t}}+\epsilon }$$

where ${\hat{m}}_{t}$ and ${\hat{v}}_{t}$ are the corrected gradient mean and variance estimates (first and second momentum respectively), while α refers to the learning rate. The model parameter at time step *t* is denoted by the variable $\theta_{t}$.

### 3.7. Grad-CAM++ for Visual Explainability

This research has employed Grad-CAM++ to enhance transparency and introduce interpretability to the DL model. Grad-CAM++ is a post hoc XAI approach that generates finer and more detailed heat maps by utilizing higher-order gradient information, making it particularly effective for complex medical images like mammograms. As a result, regions that contribute most significantly to the final prediction of the model are highlighted with greater precision.

A class-specific importance weight $\alpha_{ij}^{kc}$ is generated for each spatial location (i,j) in each feature map channel *k*. In Grad-CAM++, this is achieved by incorporating second- and third-order partial derivatives of the output $y^{c}$ with respect to the spatial features $A_{ij}^{k}$, computed as

(7) $$\alpha_{ij}^{kc}=\frac{\frac{\partial^{2}y^{c}}{{\left(\partial A_{ij}^{k}\right)}^{2}}}{2\frac{\partial^{2}y^{c}}{{\left(\partial A_{ij}^{k}\right)}^{2}}+\sum_{a,b}A_{ab}^{k}\frac{\partial^{3}y^{c}}{{\left(\partial A_{ij}^{k}\right)}^{3}}}$$

where $A_{ij}^{k}$ denotes the activation value at location (i,j) in the k−th feature map, and the derivatives represent sensitivity of the class score to the activations. Subsequently, the class activation map $L_{\mathrm{Grad}\text{-}\mathrm{CAM}++}^{c}$ is generated by aggregating the computed importance weights $\alpha_{ij}^{kc}$ and the positive gradients:

(8) $$L_{\mathrm{Grad}\text{-}\mathrm{CAM}++}^{c}=\sum_{k}\sum_{i}\sum_{j}\alpha_{ij}^{kc}\cdot \mathrm{ReLU}\left(\frac{\partial y^{c}}{\partial A_{ij}^{k}}\right)$$

Once $L_{\mathrm{Grad}\text{-}\mathrm{CAM}++}^{c}$ is computed, bilinear interpolation is applied to resize the heatmap to match the dimensions of the original input image. The final output is a color-coded overlay on the input mammogram, where brighter regions emphasize stronger influence on the model’s prediction. Such visual explanations are crucial in assisting medical professionals to verify whether the model’s decision is based on clinically meaningful features.

**Figure 4:**
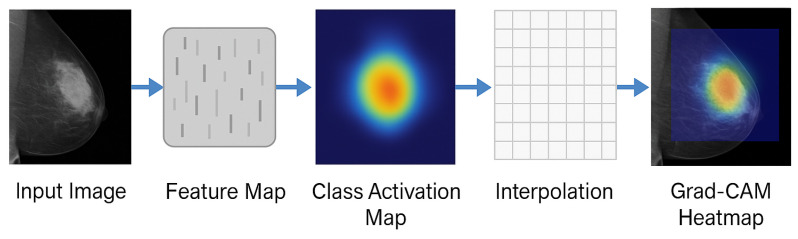
Grad-CAM++ Workflow for Class-Specific Visual Explanations.

Figure 4 illustrates the overall workflow for Grad-CAM++ visualization used in this study. Algorithm  1 discusses the proposed algorithm.
**Algorithm 1** Explainable Breast Cancer Classification Framework using EfficientNetV2 and Grad-CAM++**Input:** Three mammographic datasets: MIAS, DDSM, and InBreast**Output:** Classification predictions with explainability maps
**ForEach**(Dataset $D_{i}\in \{$MIAS, DDSM, InBreast}){ **Step 1: Preprocessing**- Convert all images to PNG format- Resize images to 224×224 using bilinear interpolation- Apply CLAHE- Perform Z-score normalization
**Step 2: Data Augmentation**- Augment dataset using rotation, flip, zoom, etc.
**Step 3: Feature Extraction and Attention**- Pass input images through EfficientNetV2 backbone- Apply CBAM for attention refinement
**Step 4: Classification**- Train a fully connected classifier head on extracted features- Use Binary Cross Entropy loss with Adam optimizer
**Step 5: Explainability**- Apply Grad-CAM++ on correctly and incorrectly predicted samples- Generate visual heatmaps to highlight lesion regions
**Step 6: Evaluation**- Assess model using Accuracy, Precision, Recall and F1-Score} **Return:** Classification performance and visual explanations for each dataset

## 4. Experimental Results and Analysis

### 4.1. Implementation Setup and Environment

The proposed architecture has been implemented in a Python environment using commonly used libraries including scikit-learn (version 1.3.2), Pandas (version 2.1.4), NumPy (version 1.26.2), Matplotlib (version 3.8.2), and LIME (version 0.2.0.1). The experimental code was created utilizing TensorFlow and Keras. The system specifications include a 64-bit architecture, a 2.40 GHz Intel(R) Core(TM) i7 processor, 16 GB of RAM, a 512 GB SSD, and an AMD Radeon GPU. All three datasets underwent preprocessing as outlined and were partitioned into 60% training and 40% testing sets, preserving the original class distribution. The splitting was performed on a patient-wise (case-level) basis within each dataset so that all mammograms from the same subject were assigned exclusively to either the training or testing set, preventing subject-level data leakage. A 10-fold cross-validation was employed to guarantee generalization and robustness. Table 4 contains the definitions and corresponding formulas for the standard classification metrics that were employed to evaluate the performance of proposed and baseline models: accuracy, precision, recall and F1-score.

### 4.2. Model Training Configuration

The model training was performed by applying the Adam optimizer with a base learning rate of η=0.0001 [48]. A batch size of B=32 and a maximum of E=50 training epochs were employed. To mitigate overfitting, early stopping was applied. The categorical cross-entropy loss function was used for multi-class classification. Mammogram images were downsized to 224×224 pixels, and data augmentation methods, including random rotation, flipping, and zooming, were employed during training. The learning rate decreased once reaching a plateau, with a patience of 5 epochs. All models underwent training via a layered train-validation-test division. The optimal model checkpoint was determined by validation accuracy and employed for the final assessment.

### 4.3. Performance Evaluation on Benchmark Datasets

Table 5 provides a comparative analysis of the outcomes of different DL models on three benchmark mammography datasets: MIAS, DDSM, and InBreast. It is clear that classic models like as VGG16 and MobileNetV2 attained reasonable accuracy and F1-scores, whereas advanced architectures like ResNet50V2 and InceptionV3 consistently surpassed them across all measures. The benchmark models were trained and evaluated with the same pre-processing methods of CLAHE enhancement, Z-score normalisation, resizing to 224×224, and identical real-time data augmentations as the new EfficientNetV2 model, thereby allowing fair and unbiased comparisons.

The enhanced performance can be credited to the architectural benefits of these models. ResNet50V2 use residual connections to alleviate vanishing gradient issues and allow deeper feature extraction. InceptionV3 utilizes multi-branch convolutional filters to extract information at various scales. This enhances the differentiation of fine-grained features in mammograms. On the other hand, MobileNetV2, being a lightweight architecture with a reduced number of parameters, is deficient in its ability to depict complex structures in medical images. VGG16, despite its increased parameter count, experiences overfitting due to the absence of modern architectural enhancements like skip connections or multi-scale processing.

However, our proposed EfficientNetV2 model considerably surpassed all baseline models across every dataset. In the MIAS dataset, a maximum accuracy of 99.85% was acquired, with a comparable high performance observed in the DDSM data sets (99.40%) and InBreast (99.70%). This shows its exceptional generalizability and robustness in various imaging settings and data set attributes. EfficientNetV2 not only performed well in classification comparison, but also proved to be computationally less expensive with merely 14 million parameters. Thus, it is a viable option for deployment in real-time clinical applications. The results presented illustrate the efficacy of our proposed design in aligning well with clinical requirements to achieve precision and efficiency.

**Figure 5:**
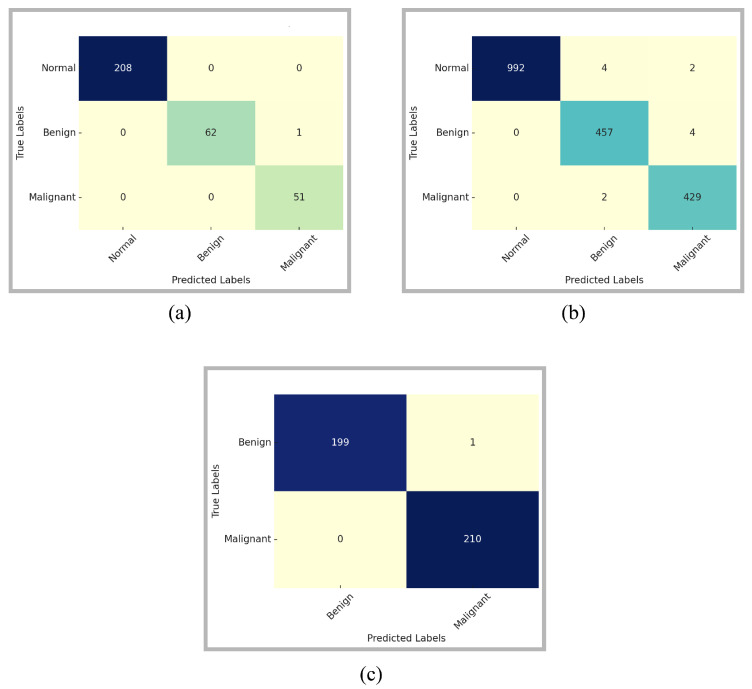
Confusion Matrices for (**a**) MIAS, (**b**) DDSM, and (**c**) InBreast using Proposed Model on full dataset before augmentation.

The class-wise performance comparison of the proposed model is provided through confusion matrices in Figure 5. The confusion matrices are computed on the full original test sets prior to online augmentation, while the corresponding class-wise performance distributions are reported quantitatively in Table 2. These matrices provide a comprehensive insight into the model’s ability to differentiate among the three classes: Normal, Benign, and Malignant. For the MIAS dataset, the model has correctly classified all Normal and Malignant cases. There is only a single Benign sample misclassified as Malignant. The model exhibited the same performance for the relatively more complex and diverse DDSM dataset. Among 992 normal samples, misclassifications were observed for only six samples. The model incorrectly classified four as Benign and two as Malignant. The model accurately distinguished the majority of Benign and Malignant instances, exhibiting only slight misunderstanding between the two categories. This suggests that although the model is remarkably reliable in distinguishing Normal samples, there exists a minor overlap in the feature representations of Benign and Malignant patterns. However, the count of misclassifications is minimal relative to the dataset size, reinforcing the model’s robustness.

The confusion matrix for the InBreast dataset further supports the generalizability of the proposed approach. There is only one Benign instance that is incorrectly identified as Malignant. Additionally, no malignant case was neglected, which is essential in practical clinical screening, as false negatives can lead to dire consequences. The considerable diagonal dominance of the matrix signifies high confidence and stability in predictions, particularly for the malignant class.

Table 6 outlines a comparative performance analysis of ResNet50V2 and the proposed EfficientNetV2 model, assessed with and without preprocessing across the three datasets. It is evident that preprocessing has substantially enhanced performance for both models in the MIAS dataset. The accuracy for ResNet50V2 has increased from 90.20% to 94.30%, whereas EfficientNetV2 reveals a significant increase from 92.50% to 99.85%, along with improvements in other parameters. This emphasizes the model’s capacity to successfully utilize improved contrast and normalized intensity distributions.

A similar pattern can be seen in the DDSM dataset, where preprocessing allows EfficientNetV2 to enhance its accuracy from 91.10% to 99.40%, in contrast to a modest improvement by ResNet50V2 (89.90% to 93.10%). Notable performance gains can be observed for the InBreast dataset that has high-resolution full-field digital mammograms. EfficientNetV2 acquires a performance improvement from 92.90% to 99.70%, and ResNet50V2 outcome increases from 91.50% to 94.50%. In all three datasets, EfficientNetV2 surpasses ResNet50V2, both with and without preprocessing, demonstrating its better architectural design, scalability, and feature learning capacity.

**Figure 6:**
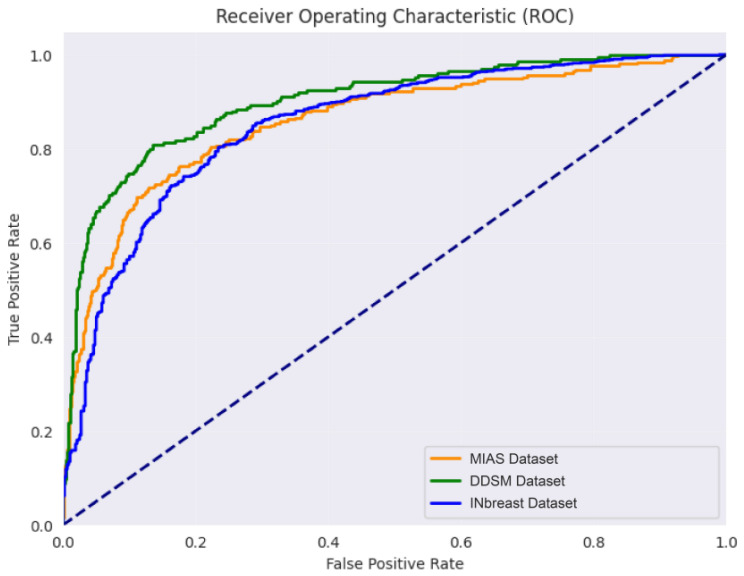
ROC curves and corresponding AUC values for the proposed EfficientNetV2-based model on the MIAS, DDSM, and InBreast test sets.

These results confirms two principal findings: (1) preprocessing techniques like CLAHE and Z-score normalization significantly enhance classification performance through enhanced structural visibility and minimizing variance among samples, and (2) the EfficientNetV2 model consistently attains state-of-the-art results, especially with high-quality preprocessing. These findings highlight the connection between modified architectural design and methodical data preparation in medical image analysis. To provide insight into the optimization dynamics, Figure 6 illustrates the evolution of training and validation accuracy and loss over the training epochs for the proposed EfficientNetV2-based model.

### 4.4. Discussion of Results

#### 4.4.1. Interpretation of Visual Explanations

The objective of this study was to improve the interpretability of CNN-based breast cancer classification by offering concise visual explanations for the classification of mammograms as normal, benign, or malignant. In clinical contexts, it is essential for explanation methods to be both consistent and time-efficient, as radiologists frequently operate under strict time constraints. Additionally, the incorporation of AI systems in healthcare practice is contingent upon the establishment of trust in these systems. Grad-CAM was the most effective interpretability technique in this context, producing stable, gradient-based visual mappings that align with model decisions and providing explanations in under one second per image (average 0.29 s). This method was found to be optimal for clinical applications, as it provided interpretable visualizations without sacrificing accuracy or rapidity.

Benign and malignant lesions are typically depicted as white or bright regions on mammographic images, which contrast with the darker breast tissue that surrounds them. Grad-CAM effectively capitalized on this distinction by creating informative heatmaps that emphasized pertinent features. The heatmaps of benign cases indicated the presence of well-defined, localized regions, which were frequently round or oval in shape. These regions appeared to be well-contained and non-invasive tissue alterations. The maps exhibited irregular, dispersed patterns in malignant cases, which were consistent with the infiltrative nature of cancerous tissue. On the other hand, regular cases lacked focused activation areas, as would be expected in healthy tissue. The reliability and relevance of Grad-CAM in breast cancer diagnostics are substantiated by the fact that these explanation patterns closely mirror real-world clinical observations.

**Figure 7:**
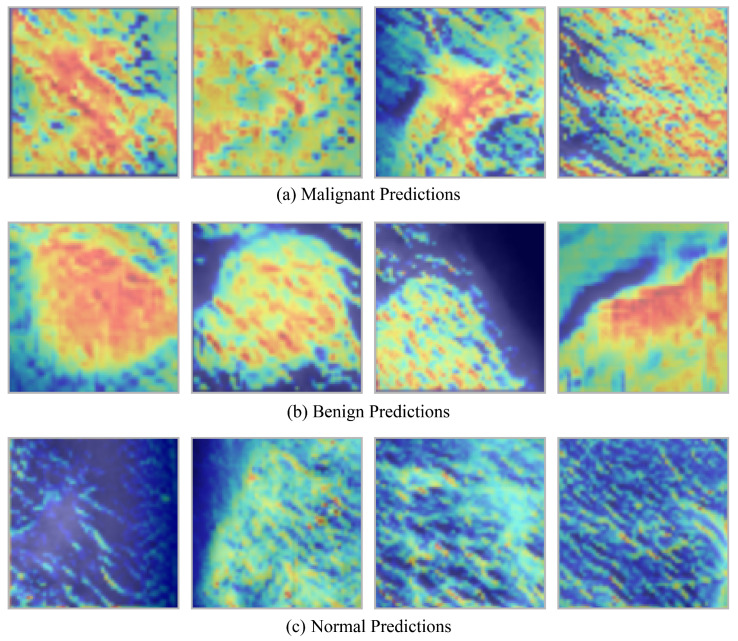
Class-Specific Grad-CAM++ Heatmaps: (**a**) Malignant, (**b**) Benign, and (**c**) Normal Cases.

Grad-CAM heatmaps generated by a CNN-based breast cancer classification model across three categories: malignant, benign, and normal tissue are depicted in Figure 7. The upper row corresponds to a malignant class, the middle row to a benign class, and the bottom row to a normal class. The decision-making process is illuminated by the heatmaps, which emphasize the regions of the mammogram that most significantly impacted the model’s prediction. The heatmaps in the upper row, which represent malignant cases, demonstrate irregular, dispersed activation patterns. These patterns are spatially dispersed and lack distinct boundaries, which implies the existence of invasive tumor structures. This visual evidence is consistent with clinical understanding, as malignant lesions frequently exhibit asymmetrical, inadequately defined masses on mammograms and infiltrate the surrounding tissue.

The middle row, which corresponds to benign cases, exhibits regions of high activation that are more localized and well-defined. Localized, non-aggressive tissue changes are frequently indicated by the heatmaps, which accentuate round or oval areas. These visual indicators are in accordance with the typical characteristics of benign findings, which are characterized by their smooth, regular borders and confined nature. In contrast, the heatmaps for normal mammograms are depicted in the bottom row. These heatmaps exhibit minimal or diffuse activations without a pronounced focus. The absence of robust activation is indicative of the absence of suspicious abnormalities, which is consistent with the presence of healthy breast tissue. In general, the figure illustrates that the model, with the assistance of Grad-CAM, effectively differentiates between normal, benign, and malignant patterns, thereby generating interpretable visual evidence that is consistent with radiological evaluations in clinical practice.

#### 4.4.2. Computational Efficiency and Time Complexity

The prediction time and build time of five DL models across the MIAS, DDSM, and InBreast datasets are presented in Table 7. Prediction time measures the average inference time per sample, while build time denotes the model training duration in seconds. Despite the fact that MobileNetV2 has the shortest build and prediction durations across all datasets due to its lightweight architecture, the results indicate that it falls short in terms of accuracy and feature learning capability, as previously discussed. Conversely, VGG16’s computational cost is not directly proportional to its performance gain, despite the fact that it has the highest build and prediction times. This is a result of its exceedingly deep architecture and complex parameter design.

The proposed EfficientNetV2 model acquires an optimal balance between classification performance and computational efficiency. It surpasses advanced models, including InceptionV3 and ResNet50V2, by maintaining acceptable build and prediction durations across all datasets. For example, EfficientNetV2 trains on the MIAS dataset in 1120 s, which is substantially faster than VGG16 (1680 s) and InceptionV3 (1520 s). Additionally, it maintains a lower prediction latency of only 0.040 s. These results substantiate the architectural efficiency of EfficientNetV2, which employs optimized convolutional blocks and compound scaling to minimize training and inference time without compromising accuracy. It is viable for deployment in time-sensitive clinical settings, where swift and precise diagnosis is critical, due to its uniform time efficiency across datasets.

As effective as they have been at achieving improved clinical performance for mammograms with the assistance of convolutional neural networks (CNN), numerous constraints remain that are shared among the deep learning methodologies currently available for mammography. The work here described continues to make use of image-level supervision (which is a characteristic feature of CNN-driven methodologies [31,32,33]), and therefore, the methodologies currently being tested here do not include any form of metadata, which could undoubtedly provide further insights into the contextual factors influencing decisions regarding mammograms. Transformer-based methodologies (e.g., ETECADx) and the residual convolutional transformer workflow have demonstrated an impressive ability to build global models of complete-field digital mammograms; however, they typically lack the explicit visual rationale needed by radiologists to interpret their findings. In contrast to this, our methodology provides the ability to build global models of complete-field digital mammograms in a more streamlined way through the design of a highly efficient CNN backbone and enhanced attention-based Grad-CAM++ prompting for visualizing features in our model. However, the degree of accuracy associated with post hoc rationalization of a model is contingent on the stability of model gradients; therefore, these models cannot be said to have provided formal causal interpretability. Lastly, while evaluation of our approach was based on three publicly accessible datasets, the validation of our methodology remains in need of external multicenter testing and clinical studies for the validation of its clinical reliability, as indicated in similar reports in the literature. Furthermore, although the proposed model exhibits consistent performance gains over state-of-the-art baselines across multiple datasets, formal statistical significance testing of these differences was not performed in the current work due to computational constraints and should be addressed in future studies. In future work, we also plan to extend this study toward a hybrid explainable federated-based vision transformer framework, inspired by [49] for breast cancer prediction using both imaging data and patient risk factors. Such a federated learning paradigm would enable multi-center collaboration without sharing raw patient data, thereby preserving privacy while improving model generalization. Integrating explainability into a federated vision transformer pipeline would also support transparent clinical decision-making across distributed healthcare environments.

#### 4.4.3. Comparison with State-of-the-Art Methods

Table 8 provides a comparative analysis of the proposed EfficientNetV2-based model in relation to other existing well-known methodologies. The chosen models encompass both hybrid CNN architectures and optimization-augmented DL variations across three benchmark datasets. The proposed model achieves an accuracy of 99.85% on the MIAS dataset, surpassing the hybrid CNN by [31] (99.17%) and the optimization-driven IMPA-ResNet50 model by [32] (98.88%). Although [33] exhibited an accuracy of 99.80% utilizing a ResNet50 integrated with Flower Pollination Optimization, the proposed approach demonstrates superior performance across all metrics, with significantly fewer parameters (about 14 M versus 25.6 M).

A maximum accuracy gain of 99.40% is attained for the DDSM dataset, surpassing BreastNet-SVM [34] (99.16%) and the hybrid CNN method [31] (98.44%). The IMPA-ResNet50 model [32] achieves an accuracy of 98.32%. The performance difference increases when compared with the optimization-enhanced ResNet model [33], whose accuracy is 93.80% accuracy. The outcomes reaffirm the robustness of the proposed architecture against more intricate data distributions. The proposed model outperforms [35] with a recall of 99.50% on the InBreast dataset. Additionally, it slightly outperforms ResNet50 in flower pollination optimization [33], demonstrating strong generalization in various clinical imaging contexts.

Broadly, when compared with existing state-of-the-art techniques, the proposed EfficientNetV2-based model shows higher classification accuracy, recall, and F1-score across all three datasets. Moreover, it has simultaneously minimized its parameter footprint. These results not only validate the model’s architectural efficacy, but highlight its practical feasibility in real-world medical imaging applications.

## 5. Conclusions

This paper introduces an explainable and computationally effective DL model for breast cancer classification from mammographic images. The model consists of EfficientNetV2 for feature extraction, the CBAM for refinement guided by attention, and Grad-CAM++ for visual interpretability. To confirm the efficacy of the proposed method, experiments were carried out on three standard mammographic datasets: MIAS, DDSM, and InBreast. The model demonstrated better performance in all metrics of evaluation, i.e., accuracy, precision, recall, and F1-score, which were consistently higher than state-of-the-art models like ResNet50V2 and InceptionV3. Significantly, CLAHE-based preprocessing and Z-score normalization significantly helped improve image quality as well as classification performance. The suggested framework, in addition to exhibiting high accuracy—achieving up to 99.85% on MIAS—also had a relatively modest parameter footprint, making practical application in clinical practice feasible. Also, the Grad-CAM++ visualizations produced class-specific and meaningful explanations that can assist radiologists in confirming and interpreting model predictions. Moreover, the model showed excellent generalizability across diverse datasets and fast training and inference times, ensuring it is viable for actual-world diagnostic use. The comparative study against other XAI methods further depicted the supremacy of the proposed approach in image-based explainability. Future research could involve expanding the model to multi-class classification, incorporating multimodal data (e.g., clinical reports, genomic profiles), and assessing model performance in future clinical trials.

## Figures and Tables

**Table 1:** Critical comparison of prior mammography-based breast cancer studies.

Study	Dataset	Model	Best Result	XAI Technique	Main Limitations
Raaj et al. [31]	MIAS, DDSM	Hybrid CNN	Acc. 99.17% (MIAS)	None	Limited interpretability, heavy architecture
Houssein et al. [32]	MIAS, DDSM	IMPA-ResNet50	Acc. 98.88% (MIAS)	None	Optimization overhead, no visual explanation
Su Rehman et al. [33]	MIAS, DDSM, InBreast	ResNet50 + FPO	Acc. 99.80% (MIAS)	None	Increased complexity, coarse feature localization
Shen et al. [35]	InBreast	All-Conv Net	Recall 86.70%	None	Modest accuracy, no attention or XAI
Raghavan et al. [36]	Infrared breast images	CNN Ensemble	Acc. >95%	Grad-CAM	No attention refinement, modality-specific
Munshi et al. [39]	Multimodal images	CNN + RF/SVM Ensemble	Acc. 96–97%	SHAP	No evaluation of spatial explanation utility

**Table 2:** Summary of mammography datasets used in this study.

Dataset	Total Images	Normal	Benign	Malignant
MIAS	322	208	63	51
DDSM	1878	998	461	431
InBreast	410	—	200	210

**Table 3:** Training and testing data split with class-wise distribution. Augmentation is applied only to the training set and does not alter test set size. Training columns show: Original → After Augmentation.

Dataset	Total	Train			Test		
		Normal	Benign	Malignant	Normal	Benign	Malignant
MIAS	322	125 → 625	38 → 190	31 → 155	83	25	20
DDSM	1878	598 → 2990	277 → 1385	252 → 1260	400	184	167
InBreast *	410	–	120 → 600	126 → 630	–	80	84

* InBreast contains no Normal class; only benign and malignant categories are available.

**Table 4:** Evaluation Metrics and Their Definitions.

Metric	Formula	Description
Accuracy	$\frac{TP+TN}{TP+TN+FP+FN}$	Ratio of total correct predictions to all predictions.
Precision	$\frac{TP}{TP+FP}$	Fraction of positive predictions that are actually correct.
Recall	$\frac{TP}{TP+FN}$	Fraction of actual positive cases correctly identified by the model.
F1-Score	$\frac{2\times \mathrm{Precision}\times \mathrm{Recall}}{\mathrm{Precision}+\mathrm{Recall}}$	Harmonic mean of Precision and Recall; balances false positives and false negatives.

**Table 5:** Performance Comparison of DL Models Across Datasets.

Dataset	DL Model	Accuracy (%)	Precision (%)	Recall (%)	F1-Score (%)	Parameters (Millions)
MIAS	VGG16	88.50	88.00	87.50	87.75	∼138 M
	ResNet50V2	90.20	89.80	89.00	89.40	∼25.6 M
	InceptionV3	91.30	91.00	90.20	90.60	∼23.9 M
	MobileNetV2	87.00	86.50	86.00	86.25	∼3.4 M
	EfficientNetV2 (Proposed)	99.85	99.80	99.70	99.75	∼14 M
DDSM	VGG16	87.60	87.20	86.40	86.80	∼138 M
	ResNet50V2	89.90	89.40	88.70	89.05	∼25.6 M
	InceptionV3	90.80	90.30	89.50	89.90	∼23.9 M
	MobileNetV2	86.20	85.70	85.00	85.35	∼3.4 M
	EfficientNetV2 (Proposed)	99.40	99.30	98.90	99.10	∼14 M
InBreast	VGG16	89.00	88.50	87.80	88.15	∼138 M
	ResNet50V2	91.50	91.00	90.20	90.60	∼25.6 M
	InceptionV3	92.40	92.00	91.30	91.65	∼23.9 M
	MobileNetV2	88.00	87.50	87.00	87.25	∼3.4 M
	EfficientNetV2 (Proposed)	99.70	99.60	99.50	99.55	∼14 M

**Table 6:** Impact of Preprocessing on Model Performance.

Dataset	Preprocessing	Model Name	Accuracy (%)	Precision (%)	Recall (%)	F1-Score (%)
MIAS	Without	ResNet50V2	90.20	89.80	89.00	89.40
	Without	EfficientNetV2 (Proposed)	92.50	92.00	91.30	91.65
	With	ResNet50V2	94.30	93.90	93.20	93.55
	With	EfficientNetV2 (Proposed)	99.85	99.80	99.70	99.75
DDSM	Without	ResNet50V2	89.90	89.40	88.70	89.05
	Without	EfficientNetV2 (Proposed)	91.10	90.60	90.00	90.30
	With	ResNet50V2	93.10	92.60	92.00	92.30
	With	EfficientNetV2 (Proposed)	99.40	99.30	98.90	99.10
InBreast	Without	ResNet50V2	91.50	91.00	90.20	90.60
	Without	EfficientNetV2 (Proposed)	92.90	92.40	91.60	92.00
	With	ResNet50V2	94.50	94.00	93.30	93.65
	With	EfficientNetV2 (Proposed)	99.70	99.60	99.50	99.55

**Table 7:** Time Complexity Analysis of Competing Models.

Dataset	Model Name	Build Time (s)	Prediction Time (s)
MIAS	VGG16	1680	0.065
	ResNet50V2	1430	0.050
	InceptionV3	1520	0.055
	MobileNetV2	820	0.030
	EfficientNetV2 (Proposed)	1120	0.040
DDSM	VGG16	1940	0.070
	ResNet50V2	1660	0.055
	InceptionV3	1750	0.058
	MobileNetV2	940	0.035
	EfficientNetV2 (Proposed)	1280	0.045
InBreast	VGG16	1810	0.068
	ResNet50V2	1570	0.053
	InceptionV3	1650	0.056
	MobileNetV2	880	0.032
	EfficientNetV2 (Proposed)	1180	0.042

**Table 8:** Comparison with Existing State-of-the-Art Methods.

Authors	Dataset	DL Model	Accuracy (%)	Precision (%)	Recall (%)	F1-Score (%)
[31]	MIAS	Hybrid CNN	99.17	—	98.00	—
[32]	MIAS	IMPA-ResNet50	98.88	—	97.61	—
[33]	MIAS	ResNet50 + Flower Pollination Optimization	99.80	—	—	—
Proposed	MIAS	EfficientNetV2 (Proposed)	99.85	99.80	99.70	99.75
[31]	DDSM	Hybrid CNN	98.44	—	97.91	—
[32]	DDSM (CBIS-DDSM)	IMPA-ResNet50	98.32	—	98.56	—
[34]	DDSM	BreastNet-SVM	99.16	—	97.13	—
[33]	DDSM (CBIS-DDSM)	ResNet50 + Flower Pollination Optimization	93.80	—	—	—
Proposed	DDSM	EfficientNetV2 (Proposed)	99.40	99.30	98.90	99.10
[35]	InBreast	All-Convolutional Network	—	—	86.70	—
[33]	InBreast	ResNet50 + Flower Pollination Optimization	99.50	—	—	—
Proposed	InBreast	EfficientNetV2 (Proposed)	99.70	99.60	99.50	99.55

## Data Availability

The implementation of this work is available at https://doi.org/10.5281/zenodo.17803282.
